# Sex Differences in Presentation and Outcomes of Transthyretin Amyloid Cardiomyopathy

**DOI:** 10.1016/j.jacadv.2026.103068

**Published:** 2026-07-28

**Authors:** Nicola Ciocca, Rubén Fuentes Artiles, Annina Studer Bruengger, Sarah Hugelshofer, Simon F. Stämpfli, Niklas F. Ehl, Lukas Pieczora, Annatina Suter, Damiano Pongan, Stefano Ministrini, Otmar Pfister, Philippe Meyer, Isaac Shiri, Lukas Hunziker, Moritz J. Hundertmark, Christoph Gräni

**Affiliations:** a Department of Cardiology, Inselspital, Bern University Hospital, University of Bern, Bern, Switzerland; b Clinic for Cardiology and Institute for Radiology and Nuclear Medicine, Zurich, Switzerland; c Department of Cardiology, Lausanne University Hospital (CHUV), University of Lausanne, Lausanne, Switzerland; d Heart Center Lucerne, Luzerner Kantonsspital, Lucerne, Switzerland and Center for Molecular Cardiology, University of Zurich, Schlieren, Switzerland; e Department of Cardiology, HOCH, Cantonal Hospital St. Gallen, St. Gallen, Switzerland; f Cardiocentro Ticino Institute, Ente Ospedaliero Cantonale, University of Italian Switzerland, Lugano, Switzerland; g Department of Cardiology, University Heart Center, University Hospital Basel, University of Basel, Basel, Switzerland; h Division of Cardiology, Geneva University Hospitals, Geneva, Switzerland

**Keywords:** ATTR-CM, cardiac amyloidosis, cardiomyopathy, gender, sex, transthyretin

## Abstract

**Background:**

Transthyretin amyloid cardiomyopathy (ATTR-CM) is more often diagnosed in men than in women but sex-specific data remain limited.

**Objectives:**

The objective of the study was to characterize sex-related differences in presentation and outcomes in patients with ATTR-CM.

**Methods:**

Consecutive prospective patients with confirmed ATTR-CM enrolled in the multicenter Swiss-CARE registry (February 2018-March 2025) were analyzed. Clinical data were obtained at diagnosis, 6 months postdiagnosis, and yearly thereafter. The primary endpoint was first major adverse cardiac event (MACE) (ie composite of heart failure hospitalization and all-cause mortality). Kaplan-Meier and Cox proportional hazards models were employed.

**Results:**

Among 567 patients with ATTR-CM (age 77 ± 7 years, 52 [9%] women), women were older at diagnosis (80.3 ± 6.1 vs 76.9 ± 6.9 years; *P* < 0.001), had a higher NYHA functional class (*P* < 0.001), higher N-terminal pro–B-type natriuretic peptide (2,574 vs 1,632 pg/mL; *P* = 0.006), and shorter 6-minute walking distance (320 ± 111 vs 411 ± 114 m; *P* = 0.001). Tafamidis was less frequently prescribed in women (50.0% vs 69.7%; *P* = 0.004). Women had a higher incidence of MACE (HR: 1.86; 95% CI: 1.13-3.04; *P* = 0.014), driven by increased heart failure hospitalizations within the first 2 years (HR: 2.21; 95% CI: 1.17-4.19; *P* = 0.015) and a trend toward higher all-cause mortality (HR: 1.82; 95% CI: 0.97-3.43; *P* = 0.06). In multivariable analyses, sex was not independently associated with MACE (*P* = 0.23).

**Conclusions:**

Women exhibited a higher risk of MACE after diagnosis; however, sex was not an independent predictor of outcomes in multivariable analysis. This disadvantage in women may be partly explained by older age at diagnosis, higher NT-proBNP levels, greater symptom burden, and a higher prevalence of comorbidities, rather than intrinsic sex-specific differences in ATTR-CM progression.

Transthyretin (TTR) amyloid cardiomyopathy (ATTR-CM), which can occur as a wild-type (ATTRwt) or hereditary (ATTRv) form, is a frequent form of cardiac amyloidosis with rapidly increasing prevalence.^1^ In contrast to the 2 most common variant forms globally (V142I, V50M), which typically present earlier in the disease course and often with predominant neurological symptoms, ATTRwt is an acquired condition that typically manifests as a gradual but nevertheless primarily progressive cardiomyopathy.^2^ ATTRwt has shifted from being a rare disease seen mainly in older individuals to a more widely recognized cause of heart failure (HF).^3^^,^^4^ This development was driven by greater physician awareness, advances in noninvasive imaging, and the availability of TTR-specific, disease-modifying therapies.^5^ Physiologically, TTR circulates as a stable tetramer. However, in ATTR-CM, destabilization of the tetramer leads to monomer dissociation and eventually aggregation of amyloid fibrils. Fibril accumulation in various tissues, specifically in the extracellular myocardial space, leads to disruption of myocardial tissues and impairs both diastolic and systolic function, ultimately resulting in overt HF and an increased burden of rhythm disorders.^6^ Following diagnosis, survival rates for untreated ATTR-CM are poor, with average survival often ranging from 3 to 5 years.^7^

Previous studies have consistently shown a marked male predominance in ATTR-CM, with men accounting for more than 80% of diagnosed cases.^8^^,^^9^ The reasons for this disparity remain elusive and may reflect different factors such as a true biological difference, underdiagnosis in women, or a combination of both. Autopsy studies have shown that ATTRwt amyloid deposits are present in up to 25% of adults over 85 years of age,^5^^,^^10^ and within these postmortem populations, the proportion of women can be as high as 40%,^11^ indicating a possible underdiagnosis in women.

Women with ATTR-CM often exhibit less left ventricular (LV) hypertrophy, which may be underestimated given that most observational studies rely on nonindexed echocardiographic measurements.^12^^,^^13^ They also tend to be older at diagnosis and may present with more advanced disease, but there are no clear differences in overall prognosis between sexes,^8^ albeit a recent analysis reported a possible association between women and higher mortality compared to men.^9^ Although multiple hypotheses involving genetic, hormonal, and diagnostic factors have been suggested, the general pathophysiological basis for the sex disparity in ATTR-CM remains incompletely understood.^8^ As both disease awareness and the availability of specific therapies have increased, understanding sex-specific differences has become increasingly important. Such insights are paramount to ensure timely and equitable diagnosis, risk stratification, and initiation of disease-specific therapy, for both men and women.

We therefore aimed to investigate clinical presentation and outcomes stratified by sex in ATTR-CM patients from the multicenter Swiss-CARE Registry. Furthermore, temporal trends were analyzed before and after 2022, as tafamidis—previously available mainly through compassionate use access—was included in the Swiss Specialties List in December 2021 enabling routine reimbursement under universal health insurance.

## Methods

### Study design and participants

In this prospective, observational analysis, patients from the Swiss Cardiac Amyloidosis Registry (Swiss-CARE; NCT04776824) with confirmed ATTR-CM were included. Patients were enrolled from seven Swiss hospitals (Inselspital, Bern University Hospital; Lucerne Cantonal Hospital; Zurich City Hospital Triemli; St. Gallen Cantonal Hospital; Lausanne University Hospital; Geneva University Hospitals; and University Hospital Basel) between February 2018 and March 2025. Diagnosis was based on positive 99mTc-DPD scintigraphy (Perugini grade 2 or 3) and/or endomyocardial biopsy. Genetic testing was offered to all patients.

### Swiss - CARE Registry

Baseline characteristics, including clinical, imaging, and genetic testing to differentiate ATTRwt and ATTRv were securely collected and managed using REDCap (Research Electronic Data Capture), a secure web-based data capture platform. Systematic follow-up data were collected after diagnosis (baseline) at 6 months and annually thereafter, capturing major adverse cardiac events (MACE) defined as a composite endpoint including hospitalization for HF and all-cause mortality. The secondary outcome was pacemaker or implantable cardioverter-defibrillator (ICD) implantation. As delayed initiation of tafamidis after diagnosis occurred in fewer than 5% of cases, patients who received tafamidis were considered as treated from the time of diagnosis for statistical analyses.

### Ethical considerations

The study was conducted in accordance with the ethical principles of the Declaration of Helsinki and all applicable national regulations. Ethical approval was obtained before data collection to ensure compliance with local and international standards (KEK Nr 2021-00135). Patient confidentiality was ensured through secure data deidentification, and data access was restricted to authorized study personnel only.

### Statistical analysis

Statistical analyses were performed using SPSS software (IBM SPSS Statistics for Macintosh, version 25.0; IBM Corp) and R (RStudio for macOS; RStudio, PBC). Continuous variables were presented as mean ± SD (if normally distributed) or median (IQR: Q1-Q3) otherwise. Differences in the distribution of continuous variables were assessed using the Mann-Whitney U test. Due to skewed distribution of N-terminal pro–B-type natriuretic peptide (NT-proBNP) values, a generalized linear model with a gamma distribution and log-link function to compare NT-proBNP levels between groups was applied. Categorical variables were expressed as counts (percentage) and their distribution between the 2 groups was analyzed using the chi-square or Fisher exact test. Survival analyses were based on Cox proportional hazards regression, providing estimated HRs with 95% CIs, and Kaplan-Meier curves, considering time to first MACE. Differences between Kaplan-Meier curves were assessed using the log-rank test. Variables with >20% missing values were excluded. For variables with ≤20% missing values, no imputation was performed, and complete-case analyses were conducted. The proportional hazards assumption was assessed using Schoenfeld residuals. In case of significant violation, time-dependent analyses were performed to account for potential changes in the effect of sex over time. Multicollinearity among covariates included in multivariable models was assessed using variance inflation factors. Patients were stratified by diagnosis date (before vs after 2022) to assess temporal trends following inclusion of tafamidis into the Swiss Specialties List in December 2021, enabling wider patient access under the current prescribing conditions. Statistical significance was defined as a *P* value <0.05.

## Results

### Baseline characteristics

Of a total of 577 patients, 567 (98%) were included in the final analysis. Five were excluded due to missing early follow-up data, 3 due to confirmed dual pathology with ATTR-CM and light chain amyloidosis, and 2 for lack of phenotypic cardiac involvement (Supplemental Figure 1). Baseline characteristics are summarized in Table 1. Fifty-two patients (ie 9%) of the cohort were women. At diagnosis, women were older than men (80.3 vs 76.9 years; *P* < 0.001) and presented with higher NYHA functional class (*P* < 0.001). Women demonstrated lower exercise capacity compared with men as measured by the 6-minute walk test (320 vs 411 m; *P* = 0.001) and higher NT-proBNP levels (2,574 vs 1,632 pg/mL; *P* = 0.006). Women were less frequently classified as stage I and more frequently as stage III according to the National Amyloidosis Centre staging system (*P* = 0.028). Comorbidities were similarly distributed between sexes, except for valvular heart disease, which was more prevalent in women (28.8% vs 17.5%; *P* = 0.044).

**Table 1:** Baseline Characteristics of Included Patients

Variables	Women (n = 52)	Men (n = 515)	*P* Value
Age at diagnosis, y	80.3 ± 6.1	76.9 ± 6.9	<0.001
Weight [kg]	67.1 ± 13.1	79.5 ± 12.9	<0.001
Height [cm]	160.9 ± 7.1	174.2 ± 6.9	<0.001
BMI [kg/m^2^]	25.9 ± 4.6	26.2 ± 3.9	0.710
NYHA functional class I	5/50 (10.0%)	160/500 (32.0%)	<0.001
NYHA functional class II	31/50 (62.0%)	287/500 (57.4%)	
NYHA functional class III	13/50 (26.0%)	48/500 (9.6%)	
NYHA functional class IV	1/50 (2.0%)	5/500 (1.0%)	
NAC I	20 (48.8%)	278 (66.8%)	0.028
NAC II	12 (29.3%)	96 (23.1%)	
NAC III	9 (22.0%)	42 (10.1%)	
6 min walking test [m]	320 ± 111	411 ± 114	0.001
NT-proBNP [pg/mL]	2,574 [900-5,252]	1,632 [814-3,016]	0.006
Tafamidis prescription	26 (50.0%)	359 (69.7%)	0.004
Perugini score	2.58 ± 0.54	2.71 ± 0.49	0.091
Comorbidities			
Carpal tunnel syndrome	18 (34.6%)	169 (32.8%)	0.792
Polyneuropathy	7 (13.5%)	53 (10.3%)	0.479
Spinal stenosis	14 (26.9%)	111 (21.6%)	0.373
Coronary artery disease	13 (25.0%)	175 (34.0%)	0.190
Valvular heart disease	15 (28.8%)	90 (17.5%)	0.044
SAVR	2 (3.8%)	17 (3.3%)	0.990
TAVI	3 (5.8%)	28 (5.4%)	0.950
AV disease > grade I	9 (20.0%)	97 (20.5%)	1.000
MV disease > grade I	16 (34.8%)	139 (29.3%)	0.546
TV disease > grade I	11 (23.4%)	108 (22.9%)	1.000
Arterial hypertension	35 (67.3%)	272 (52.8%)	0.116
Diabetes mellitus	13 (25.0%)	94 (18.3%)	0.488
Atrial fibrillation	24 (46.2%)	250 (48.5%)	0.742
History of cancer	10 (19.2%)	107 (20.8%)	0.793
History of stroke	4 (7.7%)	61 (11.8%)	0.370

AV = aortic valve; BMI = body mass index; NAC = National Amyloidosis Centre Staging System; NT-proBNP = N-terminal pro–B-type natriuretic peptide; MV = mitral valve; SAVR = surgical aortic valve replacement; TAVI = transcatheter aortic valve implantation; TV = tricuspid valve.

Among the 378 patients with available genetic testing, 95.5% had ATTRwt and 4.5% had ATTRv. The proportion of ATTRv was similar in women and men (8.3% vs 4.6%; *P* = 0.75). Echocardiography was performed in all patients (100.0%) at baseline, 99mTc-DPD scintigraphy in 550 patients (97.0%) with similar Perugini score in women and men (*P* = 0.091) and serum protein electrophoresis in 545 patients (96.1%) (Supplemental Table 1).

Baseline laboratory values showed that women had significantly lower serum creatinine than men (84.6 vs 107.8 μmol/L; *P* = 0.012), and slightly higher estimated glomerular filtration rate (eGFR) (63.1 vs 62.4 mL/min/1.73 m^2^, *P* = 0.020). The distribution of chronic kidney disease stages did not differ between sexes (*P* = 0.097) (Supplemental Table 2). The use of HF medications and oral anticoagulants at baseline was comparable between sexes, with no significant differences in anticoagulation therapy. Tafamidis was prescribed more frequently in men than in women (69.7% vs 50.0%; *P* = 0.004) (Table 1).

Echocardiography measures at baseline are summarized in Table 2. LV ejection fraction (LVEF) was predominantly preserved in both women and men, with similar mean values (55.9% vs 53.9%; *P* = 0.146). Global longitudinal strain was equally reduced in both groups (−12.3% and −12.0%; *P* = 0.953). LV end-diastolic diameter indexed values were comparable between sexes (*P* = 0.580), whereas men had higher indexed LV mass (130.9 vs 149.4 g/m^2^; *P* = 0.004). Although maximum wall thickness did not differ significantly between sexes (15.3 mm vs 16.3 mm; *P* = 0.142), values indexed to body surface area were higher in women (8.0 vs 7.0 mm/m^2^; *P* = 0.020). Left atrium was often dilated in both groups, with no significant difference (48.6 vs 47.9 mL/m^2^; *P* = 0.801).

**Table 2:** Echocardiography Measures at Baseline

	Women (n = 52)	Men (n = 515)	*P* Value
LVEF [%]	55.9 ± 10.2	53.9 ± 10.7	0.146
LV-GLS [%]	12.3 ± 4.5	12.0 ± 3.6	0.953
TAPSE [mm]	16.7 ± 4.1	17.8 ± 5.0	0.268
RVI TDI S' [cm/s]	10.0 ± 3.1	10.6 ± 2.9	0.368
DTI E' [cm/s]	4.1 ± 1.3	4.8 ± 1.7	0.032
LVEDD [mm]	41.0 ± 7.1	45.3 ± 6.9	<0.001
LVEDD indexed [mm/m^2^]	18.6 ± 11.3	20.2 ± 8.7	0.580
Maximum wall thickness [mm]	15.3 ± 2.9	16.3 ± 3.6	0.142
Max wall indexed [mm/m^2^]	8.0 ± 3.5	7.0 ± 3.6	0.020
LV-mass indexed [g/m^2^]	130.9 ± 32.7	149.4 ± 40.7	0.004
LAVI [mL/m^2^]	48.6 ± 15.9	47.9 ± 16.4	0.801
Stroke volume indexed [mL/m^2^]	31.8 ± 9.5	33.9 ± 11.8	0.378
RV/RA gradient [mmHg]	31.1 ± 10.7	30.6 ± 9.1	0.579

DTI E' = Tissue Doppler Imaging E'; LAVI = left atrium volume indexed; LV = left ventricle; LVEDD = left ventricular end-diastolic diameter; LVEF = left ventricular ejection fraction; LV-GLS = left ventricular global longitudinal strain; RA = right atrium; RV = right ventricle; RVI TDI S' = RV tissue Doppler S'; TAPSE: tricuspid annular plane systolic excursion; TDI = tissue Doppler imaging.

### Main outcomes: heart failure hospitalization and all-cause mortality

During prospective follow-up with a median duration of 2.1 (1.1-3.5) years, a total of 153 patients (27.0%) experienced MACE (composite of HF hospitalization and all-cause mortality). The secondary outcome (pacemaker or ICD implantation) occurred in 88 patients (15.5%).

Kaplan-Meier analysis demonstrated a higher incidence of MACE in women compared to men (HR: 1.86; 95% CI: 1.13-3.04; *P* = 0.014) (Central Illustration). Assessment of proportional hazards using Schoenfeld residuals indicated a time-dependent effect (*P* = 0.045), supporting the need for time-stratified analyses. Accordingly, a subanalysis restricted to the first 2 years of follow-up suggested a higher incidence of MACE in women (HR: 2.29; 95% CI: 1.30-4.04; *P* = 0.004) (Central Illustration).

**Central Illustration:**
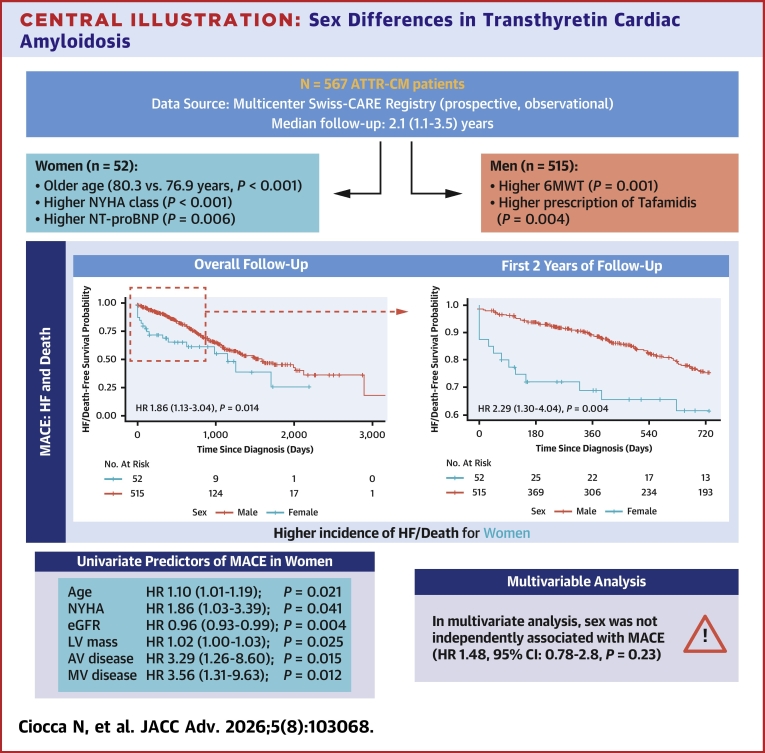
**Sex Differences in Transthyretin Cardiac Amyloidosis** 6MWT = 6 minute walk test; ATTR-CM = transthyretin amyloidosis cardiomyopathy; AV = aortic valve; eGFR = estimated glomerular filtration rate; HF = heart failure; LV = left ventricle; MACE = major adverse cardiovascular event; MV= mitral valve; NT-proBNP = N-terminal pro–B-type natriuretic peptide.

**Figure 1:**
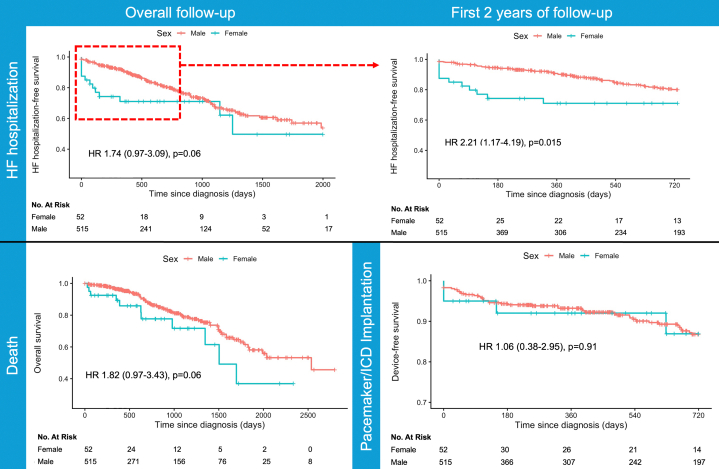
**HF Hospitalization, Death, and Pacemaker/ICD Incidence Over Time for Women and Men** Kaplan-Meier curves showing HF hospitalization, death, and pacemaker/ICD incidence over time for women and men, unadjusted. HF = heart failure; ICD = implantable cardioverter-defibrillator.

To further dissect the individual components of the composite endpoint, sensitivity analyses were performed. Over the entire follow-up, there was a trend toward a higher risk of HF hospitalization in women (HR: 1.74; 95% CI: 0.97-3.09; *P* = 0.06), with a significantly higher incidence observed within the first 2 years (HR: 2.21; 95% CI: 1.17-4.19; *P* = 0.015) (Figure 1). Women also exhibited a trend toward higher all-cause mortality during overall follow-up (HR: 1.82; 95% CI: 0.97-3.43; *P* = 0.06) (Figure 1).

There was no significant difference between women and men in the incidence of the secondary composite outcome of pacemaker or ICD implantation (HR: 1.06; 95% CI: 0.38-2.95; *P* = 0.91) (Figure 1).

### Predictors of heart failure hospitalization and all-cause mortality

The main variables associated with MACE (see Table 3), were higher age at diagnosis (*P* < 0.001 overall and for men; *P* = 0.021 for women), female sex (*P* = 0.014), NYHA functional class (*P* < 0.001 overall and for men, *P* = 0.041 for women), tafamidis use (*P* < 0.001 overall), history of atrial fibrillation (*P* < 0.001 overall and for men, *P* = 0.72 for women), higher NT-proBNP (*P* < 0.001 overall and for men, *P* = 0.19 for women), lower eGFR (*P* < 0.001 overall and for men, *P* = 0.004 for women), lower hemoglobin (*P* < 0.001 overall and for men, *P* = 0.13 for women), lower LVEF (*P* < 0.001 overall and for men, *P* = 0.09 for women), presence of tricuspid (*P* < 0.001 overall and for men, *P* = 0.001 for women), aortic (*P* < 0.001 overall, *P* = 0.002 for men, *P* = 0.015 for women), or mitral valve disease (*P* < 0.001 overall, *P* = 0.005 for men, *P* = 0.012 for women). Higher indexed LV mass, greater wall thickness, and higher indexed wall thickness were associated with MACE in women, but not in the overall population or in men. The association between tafamidis and MACE in women was not assessed due to the limited number of patients.

**Table 3:** Univariate and Multivariable Association Between Clinical, Laboratory, and Imaging Variables and the Incidence of MACE in the Overall Population and in the Women and Men Subgroups

	All Patients		Female		Male		Multivariable for all Patients	
	HR 95% CI	*P* Value	HR 95% CI	*P* Value	HR 95% CI	*P* Value	HR 95% CI	*P* Value
Age at diagnosis	1.09 (1.06-1.12)	<0.001	1.10 (1.01-1.19)	0.021	1.09 (1.06-1.12)	<0.001	1.05 (1.01-1.09)	0.005
Female	1.86 (1.13-3.04)	0.014					1.48 (0.78-2.80)	0.23
BMI [kg/m^2^]	0.96 (0.92-1.00)	0.06	0.95 (0.82-1.09)	0.46	0.96 (0.92-1.01)	0.10	0.99 (0.94-1.04)	0.62
NYHA functional class	1.89 (1.51-2.37)	<0.001	1.86 (1.03-3.39)	0.041	1.84 (1.42-2.37)	<0.001	1.04 (0.76-1.42)	0.80
Arterial hypertension	1.38 (0.99-1.92)	0.06	3.91 (0.90-17.03)	0.07	1.24 (0.88-1.76)	0.22		
History of valvular heart disease	1.66 (1.17-2.37)	0.005	2.05 (0.78-5.37)	0.14	1.54 (1.05-2.26)	0.026		
History of coronary artery disease	1.36 (0.98-1.88)	0.07	2.31 (0.89-6.00)	0.08	1.32 (0.94-1.87)	0.11		
Pacemaker implanted	1.44 (0.93-2.23)	0.10	1.53 (0.43-5.42)	0.51	1.45 (0.91-2.31)	0.12		
Tafamidis	0.41 (0.29-0.57)	<0.001	a		0.41 (0.29-0.60)	<0.001	0.66 (0.43-1.02)	0.06
History of atrial fibrillation	1.90 (1.37-2.64)	<0.001	1.19 (0.45-3.16)	0.72	2.02 (1.42-2.87)	<0.001		
Obstructive sleep apnea	0.82 (0.50-1.32)	0.41	0.50 (0.11-2.34)	0.38	0.87 (0.52-1.45)	0.59		
NT-proBNP (per 500 ng/mL increase)	1.06 (1.04-1.07)	<0.001	1.03 (0.99-1.07)	0.19	1.06 (1.04-1.07)	<0.001	1.03 (1.00-1.05)	0.043
eGFR [mL/min/1.73 m^2^]	0.98 (0.97-0.99)	<0.001	0.96 (0.93-0.99)	0.004	0.98 (0.97-0.99)	<0.001	0.99 (0.98-1.00)	0.041
Creatinine [umol/L]	1.00 (1.00-1.00)	0.05	1.01 (1.00-1.02)	0.011	1.00 (1.00-1.00)	0.11		
Hemoglobin [g/L]	0.98 (0.97-0.99)	<0.001	0.97 (0.92-1.01)	0.13	0.98 (0.98-0.99)	<0.001	1.00 (0.99-1.01)	0.53
LVEF [%]	0.97 (0.96-0.98)	<0.001	0.96 (0.92-1.01)	0.09	0.97 (0.96-0.99)	<0.001	0.98 (0.96-1.00)	0.022
TAPSE [mm]	0.90 (0.86-0.94)	<0.001	0.96 (0.85-1.09)	0.51	0.90 (0.86-0.93)	<0.001		
LV global longitudinal strain [%]	1.12 (1.05-1.19)	<0.001	1.10 (0.97-1.26)	0.14	1.12 (1.05-1.20)	0.001		
Stroke volume indexed [mL/m^2^]	0.97 (0.95-0.99)	0.004	0.96 (0.89-1.03)	0.25	0.97 (0.95-1.00)	0.018		
LAVI [mL/m^2^]	1.01 (1.00-1.02)	0.012	0.99 (0.95-1.03)	0.65	1.01 (1.00-1.03)	0.008		
TV regurgitation > grade I	2.28 (1.64-3.18)	<0.001	7.36 (2.21-24.49)	0.001	2.17 (1.53-3.09)	<0.001		
RV/RA gradient	1.02 (1.00-1.04)	0.05	1.04 (0.98-1.10)	0.21	1.01 (0.99-1.04)	0.17		
Aortic valve disease > grade I	1.89 (1.35-2.65)	<0.001	3.29 (1.26-8.60)	0.015	1.75 (1.22-2.51)	0.002		
Mitral valve disease > grade I	1.82 (1.31-2.54)	<0.001	3.56 (1.31-9.63)	0.012	1.67 (1.17-2.38)	0.005		
LV mass indexed	1.00 (1.00-1.00)	0.84	1.02 (1.00-1.03)	0.025	1.00 (1.00-1.00)	0.91		
Maximum wall thickness	1.00 (0.96-1.05)	0.90	1.34 (1.06-1.68)	0.015	0.99 (0.94-1.05)	0.82		
Maximum wall thickness indexed	1.05 (0.97-1.15)	0.22	1.69 (1.19-2.39)	0.003	1.01 (0.92-1.10)	0.90		
Perugini score	1.47 (0.99-2.19)	0.05	1.74 (0.63-4.79)	0.29	1.55 (1.00-2.42)	0.05		

eGFR = estimated glomerular filtration rate; other abbreviations as in Tables 1 and 2.

a Model not estimable and unstable due to low number of patients.

In multivariable analyses, independent predictors of MACE were older age at diagnosis (*P* = 0.005), higher NT-proBNP (*P* = 0.043), lower eGFR (*P* = 0.041), and lower LVEF (*P* = 0.022), whereas female sex was not independently associated with MACE (*P* = 0.23) (Table 3).

### Temporal improvement in diagnosis

Baseline characteristics stratified by time of diagnosis (before and after January 2022, when tafamidis was included in the Swiss Specialties List, enabling wider patient access under universal health insurance) are reported in Table 4. Age at diagnosis did not differ significantly between patients diagnosed before and after 2022, in either women (*P* = 0.117) or men (*P* = 0.191). However, women were significantly older than men at the time of diagnosis both before and after 2022 (before 2022: 82.2 ± 5.1 vs 77.5 ± 7.1; *P* = 0.002; after 2022: 79.6 ± 6.6 vs 76.5 ± 6.8 years; *P* = 0.010). Both women and men diagnosed after 2022 had lower NT-proBNP levels (*P* < 0.001 for women, *P* = 0.002 for men) compared with patients of the same sex diagnosed before 2022, along with lower NYHA functional class (*P* = 0.026 for women, *P* = 0.004 for men). However, women diagnosed after 2022 still had a higher NYHA functional class compared with men (2.0 ± 0.6 vs 1.7 ± 0.6; *P* = 0.005), whereas NT-proBNP levels were comparable between groups (2,114 vs 1,556 pg/mL; *P* = 0.363). Within each sex, tafamidis prescription did not differ before and after 2022 (*P* = 0.254 for women and *P* = 0.089 for men). In addition, men diagnosed after 2022 showed better longitudinal strain values (*P* = 0.006) and less pronounced LV hypertrophy (*P* < 0.001) than those diagnosed before 2022.

**Table 4:** Baseline Characteristics of Women and Men, Stratified by Year of Diagnosis (Before vs After January 2022)

Variables	Women			Men		
	Before 2022	After 2022	*P* Value	Before 2022	After 2022	*P* Value
Age at diagnosis, y	82.2 ± 5.1	79.6 ± 6.6	0.117	77.5 ± 7.1^a^	76.5 ± 6.8^a^	0.191
NT-proBNP [pg/mL]	3,520 [1,389-9,190]	2,114 [834-3,918]	<0.001	1806 [984-4,003]^a^	1,556 [730-2,864]	0.002
NYHA functional class	2.5 ± 0.7	2.0 ± 0.6	0.026	1.9 ± 0.7^a^	1.7 ± 0.6^a^	0.004
LVEF [%]	55.1 ± 11.1	56.4 ± 9.8	0.894	53.4 ± 11.2	54.1 ± 10.4	0.641
LV-GLS [%]	−11.8 ± 5.5	−12.6 ± 3.9	0.578	−11.4 ± 3.7	−12.4 ± 3.5	0.006
LV-mass indexed [g/m^2^]	137 ± 38.7	126.3 ± 27.5	0.622	158.6 ± 43.6	143.7 ± 37.7	<0.001
Tafamidis	12/20 (60.0%)	14/32 (43.8%)	0.254	141/190 (74.2%)	218/325 (67.1%)^a^	0.089

*P* values refer to within-sex comparisons between the periods before and after 2022.

Abbreviations as in Tables 1 and 2.

a Indicates a statistically significant difference compared with the corresponding value in women within the same time period.

## Discussion

In this multicenter ATTR-CM cohort study, women experienced a significantly higher incidence of HF hospitalization and a trend toward higher mortality compared with men, particularly within the first 2 years of follow-up. This early disadvantage was associated with older age and more advanced disease burden at presentation, as reflected by higher National Amyloidosis Centre stage, NYHA functional class, NT-proBNP levels, and reduced functional capacity (6-minute walk test). In addition, women were less frequently treated with tafamidis, which may have further contributed to their increased early risk. Nevertheless, after adjustment for major confounders, sex was not independently associated with outcomes in multivariable analyses. Taking into account the limited number of women included, this finding supports the hypothesis that once differences in age and disease severity at the time of diagnosis are accounted for the subsequent clinical course of ATTR-CM appears similar regardless of sex.^14^ Results from the temporal analysis seem to indicate a lower disease severity at diagnosis over time in both women and men; however, women continued to present with a higher disease burden than men, even among more recently diagnosed patients. Thus, our data suggest that sex-related differences in ATTR-CM may be, at least partly, related to delayed diagnosis and more advanced disease stages in women, rather than purely reflecting intrinsic biological differences. This finding is consistent with existing evidence and reflects patterns observed across a broad spectrum of cardiovascular diseases, in which women tend to seek medical care later, are older at presentation, and present with more advanced disease at the time of initial evaluation compared with men.^8^^,^^15^, ^16^, ^17^

Moreover, imaging parameters were largely similar between women and men in our cohort, suggesting that comparable measurements may still require sex-specific interpretation, particularly in light of the increased early risk of MACE observed in women. Our analysis showed that LV mass (including LV mass index and indexed maximum wall thickness) were associated with MACE only in the female subgroup, but not in men. This observation highlights the potential need to revisit current diagnostic thresholds and imaging red flags, which may not fully capture disease severity in women and could benefit from a sex-specific approach. Consistent with this, Patel et al^12^ reported no significant sex-related differences in ATTR-CM progression by echocardiography at 1 year and highlighted that sex-specific indexing of echocardiographic parameters, together with improved inclusion of women in clinical studies, is essential to avoid the misconception of a milder phenotype in women. The absence of systematic cardiac magnetic resonance imaging data represents an additional limitation, as parameters such as extracellular volume, native T1, and late gadolinium enhancement may provide complementary insights into myocardial tissue characteristics and could be particularly relevant when investigating sex-related differences in disease burden.

Women presented with more comorbidities, which indeed appear to play a particularly important role in this population. This is not only observed in ATTR-CM but also in HF with preserved ejection fraction.^18^ Despite inclusion of a geographically representative cohort of Swiss patients in our multicenter registry, women remained under-represented. This under-representation may partly be due to a lower prevalence of ATTR-CM in women, but a further component could be potential underdiagnosis and observation bias. Whether another possible explanation relates to hormonal influences remains unclear. Hormonal differences have been proposed to affect disease expression, particularly in ATTRv, with some studies suggesting that premenopausal women may have partial protection against cardiac involvement.^19^ Given the reduced number of both ATTRv cases and premenopausal women in our cohort, we were unable to explore this hypothesis further.

In our cohort, despite the limited number of women, tafamidis was prescribed less frequently. This lower prescription rate may have different reasons: firstly, it could be related to more advanced disease stages among women, conditions in which tafamidis may have limited therapeutic benefit and, given its high cost, may not have been approved or considered appropriate for initiation. Secondly, older age and greater frailty at diagnosis may discourage clinicians from initiating a long-term, expensive therapy with delayed clinical benefit. No study has specifically investigated sex-related differences in disease progression or treatment response to tafamidis in men vs women and addressing this important question exceeds the statistical power of our analysis. The ATTR-ACT trial, which led to the approval of tafamidis, included over 440 ATTR-CM patients, of whom only approximately 10% were women and no sex-specific subanalysis of outcomes was performed.^5^ Nevertheless, clinical experience to date supports the efficacy of tafamidis in both men and women.

Clinically, our study emphasizes the importance of recognizing newly diagnosed women with ATTR as a high-risk group for early MACE, largely driven by older age, more advanced disease stage, and higher burden of comorbidities. These findings highlight the need for increased awareness in the early disease stages. Our temporal analyses suggest an improvement in ATTR-CM recognition over time, reflected by a reduction in age at diagnosis in both women and men and, more importantly, by diagnosis at earlier disease stages. In patients diagnosed after 2022, we observed an attenuation of sex-related disparities, as shown by comparable NT-proBNP levels at diagnosis. Nevertheless, residual differences persist, and women remain older at diagnosis with a higher symptom burden. Given the biological differences between men and women, it is conceivable that some diagnostic red flags, particularly features relating to initial imaging tests such as echocardiography, may differ between women and men and require further investigation and sex-specific adaptation. Importantly, the lower prescription rate of tafamidis in women, even among the most recently diagnosed patients, warrants particular attention and highlights a potential treatment disparity that should be carefully considered in clinical practice.

Future research should aim to include more women, explore which specific comorbidities most strongly impact outcomes and define sex-specific diagnostic thresholds and imaging red flags. Such efforts are critical to improve sex-specific management strategies and optimizing care for women with cardiac amyloidosis.

### Study Limitations

Despite its strength our study has limitations that should be considered when interpreting the findings. Firstly, the relatively small number of women in our cohort represents an important limitation and may have reduced the statistical power to detect sex-specific differences. Secondly, patients with ATTRv were under-represented in our cohort. Therefore, the conclusions of this study may not equally apply to the ATTRv population. Thirdly, this study relied exclusively on echocardiographic parameters for structural and functional cardiac assessment. Although echocardiography is widely available and clinically relevant, the lack of complementary imaging modalities—such as cardiac magnetic resonance or scintigraphy—precluded a more comprehensive evaluation of myocardial amyloid burden and tissue characterization. Finally, genetic testing was unavailable in approximately 30% of patients; this primarily reflects very early included cases, in whom genetic testing was not always pursued, as well as a small number of recently diagnosed patients in whom test results are pending.

## Conclusions

In this observational multicenter ATTR-CM analysis, women experienced a significantly higher incidence of HF hospitalization and a trend toward higher mortality compared with men. Nevertheless, after adjustment for major confounders, sex was not independently associated with outcomes. This early disadvantage in women appears to be mainly attributable to older age and more advanced disease stages at diagnosis rather than intrinsic sex-specific differences. Our findings underscore the importance of early diagnosis, comprehensive comorbidity management, and the investigation of sex-specific diagnostic thresholds and imaging red flags. Enhancing representation of women in clinical trials remains essential to better understand sex-specific disease patterns and optimize outcomes in cardiac amyloidosis.

Perspectives **COMPETENCY IN MEDICAL KNOWLEDGE:** Women with ATTR-CM represent a higher risk and undertreated subgroup in the early phase after diagnosis, highlighting the importance of timely detection and comprehensive clinical assessment.

**TRANSLATIONAL OUTLOOK:** Efforts to develop sex-specific diagnostic criteria and optimize access to disease-modifying therapies may help reduce outcome disparities in ATTR-CM.

## Funding support and author disclosures

The Swiss-CARE Registry is funded by institutional grants to the University Hospital Bern by Pfizer, AstraZeneca, Alnylam, and Bayer. The sponsors of the registry had no role in the study design, data collection, analysis, interpretation, or manuscript writing. Dr Gräni has received funding from the Swiss National Science Foundation, InnoSuisse, Center for Artificial Intelligence in Medicine University Bern, GAMBIT foundation, Novartis Foundation for Medical-Biological Research, and Swiss Heart Foundation, outside of the submitted work. Dr Hundertmark is supported by a Protected Research Time grant by the Medical Faculty of the University of Bern, Switzerland. Dr Hundertmark has participated in educational events and advisory boards for Alnylam, AstraZeneca, Bayer Healthcare, and Pfizer with compensation paid to the institution. Dr Meyer participated in advisory boards and seminars organized by AstraZeneca, Bayer, Boehringer Ingelheim, BMS, and Pfizer. All honoraria received for these activities were entirely paid since 2015 to the GEcor Foundation, a private research foundation affiliated with the cardiology division of the Geneva University Hospitals. Dr Hugelshofer participated in advisory boards and symposia organized by AstraZeneca, Alnylam, Bayer, and Pfizer. All honoraria received for these activities were paid to the Lausanne University Hospital. Dr Bruengger participated in advisory boards and symposia organized by Alnylam, Bayer, BMS, and Pfizer. All honoraria received for these activities were paid to the research foundation of Stadtspital Zurich. Dr Ehl participated in advisory boards and symposia organized by Alnylam, AstraZeneca, Bayer, BMS, Novartis, and Pfizer. Dr Stämpfli reports travel grants, speaker fees, consulting fees, and proctoring fees from Abbott Structural Heart, Alnylam, Amgen, AstraZeneca, Bayer, Bristol-Myers Squibb, Daiichi Sankyo, Edwards Lifesciences, Fumedica, Novartis, Novo Nordisk, Polares Medical, Pfizer, and Takeda. Dr Pfister participated in advisory boards organized by AstraZeneca, Alnylam, Bayer, Boehringer Ingelheim, BMS, Eli Lilly, MSD, Novo Nordisk, and Pfizer. All honoraria received for these activities were entirely paid to research fonds affiliated with the cardiology division of the University Hospital Basel. All other authors have reported that they have no relationships relevant to the contents of this paper to disclose.
