# Supplementary material for: Sex Differences in Presentation and Outcomes of Transthyretin Amyloid Cardiomyopathy
Source: JACC Adv. 2026 Jul 28;5(8):103068. doi: 10.1016/j.jacadv.2026.103068 (PMC13430163; doi:10.1016/j.jacadv.2026.103068)
Supplement: Supplemental_Material [file mmc1.pdf]

SUPPLEMENTARY MATERIALS

Test performed at diagnosis	N (%)
**Scintigraphy**	550 (97.0%)
**Genetic analysis**	378 (66.7%)
**Serum electrophoresis**	545 (96.1%)
**Endomyocardial biopsy**	31 (5.5%)
**Bone marrow biopsy**	21 (3.7%)

Supplementary Table 1: Diagnostic test performed at baseline.

Laboratory testing	Women	Men	P-Value
**Sodium [mmol/l]**	139.4 +/- 2.2	140.2 +/- 2.5	0.206
**Potassium [mmol/l]**	4.1 +/- 0.4	4.2 +/- 0.4	0.165
**Creatinine [umol/l]**	84.6 +/- 28.8	107.8 +/- 38.6	0.012
**eGFR [ml/min/1.73m^2]**	63.1 +/- 17.4	62.4 +/- 17.5	0.020
**CKD KDIGO Stage 1**	1.9%	4.1%	0.097
**CKD KDIGO Stage 2**	34.6%	46.2%	
**CKD KDIGO Stage 3a**	19.2%	21.0%	
**CKD KDIGO Stage 3b**	13.5%	10.7%	
**CKD KDIGO Stage 4**	13.5%	4.3%	
**CKD KDIGO Stage 5**	0.0%	0.2%	
**Troponin T-hs [ng/l]**	42.0 +/- 21.9	50.3 +/- 30.4	0.682
**NT-proBNP [pg/ml]**	2574 [900-5252]	1632 [814-3016]	0.006
**HbA1c [%]**	6.2 +/- 0.9	6.0 +/- 0.7	0.890
**Hemoglobin [g/l]**	132.8 +/- 9.0	140.4 +/- 18.2	<0.001
**Hematocrit**	0.40 +/-0.03	0.41 +/-0.05	0.003
**Ferritin [ug/l]**	159.7 +/- 99.8	215.8 +/- 180.9	0.040
**Transferrin Saturation [%]**	25.4 +/- 8.7	24.7 +/- 10.3	0.882

Supplementary Table 2: laboratory exams performed at baseline. eGFR: estimated glomerular filtration rate (EPI-CKD). HCT: hematocrit.

**Medications**	**Women**	**Men**	**P-Value**
**Disease modifying therapy**			
Tafamidis	26 (50.0%)	359 (69.7%)	0.004
**Heart failure medication**			
ACE-Inhibitor	12 (23.1%)	118 (22.9%)	0.979
ARB	16 (30.8%)	137 (26.6%)	0.519
Entresto	2 (3.8%)	31 (6.0%)	0.524
Betablocker	24 (46.2%)	241 (46.8%)	0.930
MRA	11 (21.2%)	108 (21.0%)	0.975
SGLT2-Inhibitor	14 (26.9%)	140 (27.2%)	0.968
Loop diuretics	36 (69.2%)	276 (53.6%)	0.031
**Anticoagulation**			
Vitamin K antagonist	1 (1.9%)	23 (4.5%)	0.385
Rivaroxaban	7 (13.5%)	119 (23.1%)	0.111
Eliquis	13 (25.0%)	101 (19.6%)	0.356
Edoxaban	0 (0.0%)	17 (3.3%)	0.183
Dabigatran	1 (1.9%)	2 (0.4%)	0.146
**Other**			
Amiodarone	5 (9.6%)	52 (10.1%)	0.912

Supplementary Table 3: medication at baseline and Tafamidis prescription among men and women. MRA: Mineralocorticoid Receptor Antagonist. SGLT2: Sodium-glucose cotransporters.

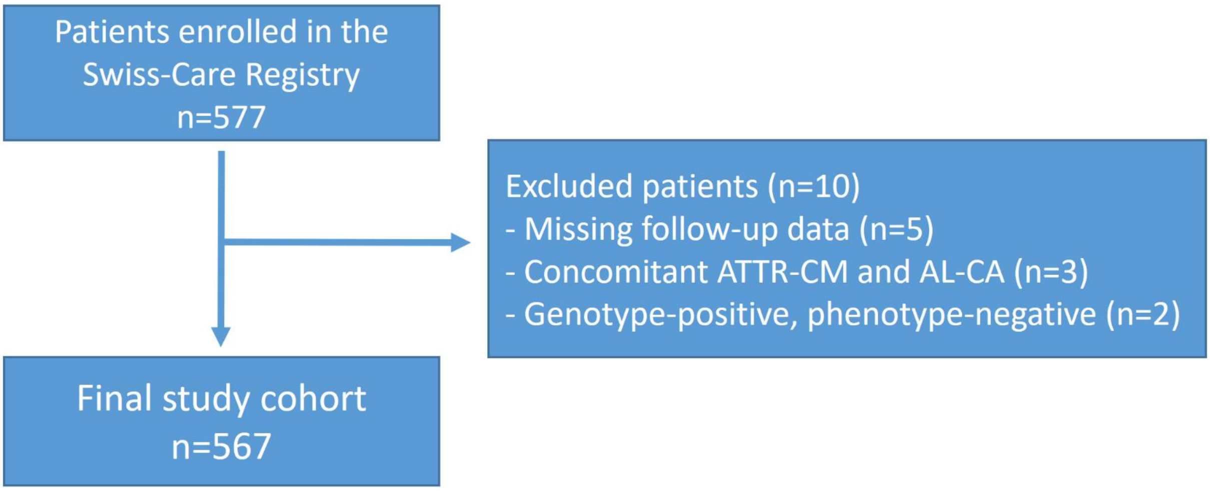

Supplementary Figure 1: Flowchart of patient selection and study population.
